# Social anxiety and MDMA-assisted therapy investigation: a novel clinical trial protocol

**DOI:** 10.3389/fpsyt.2023.1083354

**Published:** 2023-07-14

**Authors:** M. Kati Lear, Sarah M. Smith, Brian Pilecki, Chris S. Stauffer, Jason B. Luoma

**Affiliations:** ^1^Portland Psychotherapy Clinic, Research, and Training Center, Portland, OR, United States; ^2^Department of Psychiatry, Oregon Health and Science University, Portland, OR, United States

**Keywords:** MDMA, social anxiety disorder, psychedelic research, clinical trial protocol, psychedelic assisted therapy

## Abstract

**Background:**

Social anxiety disorder (SAD) is a serious and prevalent psychiatric condition that heavily impacts social functioning and quality of life. Though efficacious treatments exist for SAD, remission rates remain elevated and a significant portion of those affected do not access effective treatment, suggesting the need for additional evidence-based treatment options. This paper presents a protocol for an open-label pilot study of MDMA-assisted therapy (MDMA-AT) for social anxiety disorder. The study aims to assess preliminary treatment outcomes, feasibility and safety, and psychological and physiological processes of change in the treatment of SAD with MDMA-AT. A secondary aim includes the development of a treatment manual for MDMA-AT for SAD.

**Method:**

The outlined protocol is a randomized, open-label delayed treatment study. We will recruit 20 participants who meet criteria with moderate-to-severe social anxiety disorder (SAD) of the generalized subtype. Participants will be randomly assigned to an immediate treatment (*n* = 10) or delayed treatment condition (*n* = 10). Those in the immediate treatment condition will proceed immediately to active MDMA-AT consisting of three preparation sessions, two medicine sessions in which they receive oral doses of MDMA, and six integration sessions over approximately a 16-week period. The delayed treatment condition will receive the same intervention after a 16-week delay. Our primary outcome is SAD symptom reduction as measured by the Liebowitz Social Anxiety Scale administered by blinded raters at post-treatment and 6 month follow up. Secondary outcomes include changes in functional impairment, feasibility and safety measures, and novel therapeutic processes of change including shame and shame-related coping, belongingness, self-concealment, and self-compassion at post-treatment. Exploratory outcomes are also discussed.

**Discussion:**

The results of this pilot trial advance the field’s understanding of the acceptability and potential effectiveness of MDMA-AT for social anxiety disorder and provide an overview of relevant therapeutic mechanisms unique to SAD. We hope findings from this protocol will inform the design of subsequent larger-scale randomized controlled trials (RCT) examining the efficacy of MDMA-AT for SAD.

**Clinical trial registration:**

https://clinicaltrials.gov/, NCT05138068.

## 1. Introduction

Social anxiety disorder (SAD) is a prevalent and disabling psychiatric disorder that profoundly influences the lives of those affected. In the United States, recent estimates showed 7.4% of adults met criteria for SAD in the past year and approximately 13% will experience SAD at some point in their lives (1). SAD is characterized by intense fear of being negatively evaluated by others in social situations. People with SAD report strong feelings of distress in anticipation of and during social situations and this fear is often accompanied by inaccurate perceptions of others’ overly critical attitudes and high social standards for performance (2).

SAD commonly precedes the development of other psychiatric conditions. For instance, those diagnosed with SAD significantly increases one’s risk of developing major depressive disorder (3) and doubles their likelihood of developing an alcohol use disorder (4). People with SAD are also significantly more likely to experience suicidal ideation (SI) than those diagnosed with other anxiety disorders (5, 6). In addition to substantial psychological suffering, SAD is associated with marked functional impairment characterized by reports of reduced quality of life and lower socioeconomic status (7, 8) as well as significant public health costs including increased unemployment, workplace absenteeism, and lower worker productivity (9, 10).

People tend to develop SAD in adolescence and the disorder usually assumes a chronic course if it remains untreated (1, 11). A recent report of the World Health Organization World Mental Health Survey (7) gathered across 27 high- (*n* = 16) and low- and middle-income countries (*n* = 11) from respondents with a lifetime diagnosis of SAD (*n =* 5,686; total respondents: *N =* 117,856) showed that only one in five had *ever* received treatment for their social anxiety, underscoring the scope of the global impact of untreated SAD. Furthermore, those who successfully access treatment may have to make several attempts before finding helpful treatments (7), which may present special challenges for those with SAD due to characteristic social avoidance.

Current evidence-based therapies for SAD include medications and psychotherapy, but a significant proportion of patients either do not respond or remain considerably symptomatic at the end of treatment (12, 13). In terms of psychotherapy, individual and group-based cognitive-behavioral therapy (CBT) that incorporates systematic exposure to social stimuli demonstrates the strongest outcomes (13). Meta-analytic evidence examining the efficacy of CBT for SAD suggests medium-large treatment effects for individual [*Hedges g* = 0.77, 95% CI (0.44, 1.11)–0.87, 95% CI (0.51–1.23) (14)] and group-based CBT [*Hedges’ g* = 0.84, 95% CI (0.72; 0.97) (15)] compared to control conditions following treatment. Effect sizes at long-term follow up appear attenuated, with small-moderate differences in SAD symptoms associated with CBT relative to control conditions at 1–6 month follow up [*Hedges*’ *g* = 0.60, 95% CI (0.36–0.85)] through follow up extending to 12 months or more [*Hedges’ g* = 0.42, 95% CI (0.04–0.79) (16)]. Despite being an effective frontline treatment that results in symptom reduction for most clients, meta-analytic evidence shows that many who receive CBT do not achieve remission (17) and some estimates of group-based CBT for SAD suggest nontrivial dropout rates (18). Taken together, these findings suggest that CBT may not be a preferred or feasible treatment for everyone affected by SAD. For pharmacotherapy, meta-analytic evidence supports the conclusion that the broad drug class of SSRIs and SNRIs are superior to placebo [*Standardize Mean Difference* = 0.55, 95% CI (0.49, 0.60) (19)], but also that SSRIs are associated with higher treatment dropout than placebo over the treatment course (20) and ongoing treatment incurs side effects.

Thus, there is a need to develop innovative, durable, and brief interventions that could alleviate suffering and enhance the quality of life of those with SAD. To this end, there has been growing interest in the use of psychopharmacological interventions to enhance behavioral interventions for SAD. One potentially promising pharmacological intervention that may enhance psychotherapy outcomes is 3,4-methylenedioxymethamphetamine (MDMA), which is explored in this paper.

### 1.1. Methylenedioxymethamphetamine and MDMA-assisted therapy

MDMA, or 3,4-methylenedioxymethamphetamine, was first synthesized by Merck in 1912 and was discovered by therapists as an adjunct to psychotherapy in the late 1970s (21). Early claims suggested that MDMA facilitated feelings of empathy, increased self-awareness, reduction in psychological defenses, and increased communication and connection with others (21, 22). Although MDMA showed potential as a tool in therapeutic treatment settings, it was banned in the United States in 1985 after it became more widely used in recreational settings, and eventually became banned in most other countries as well (23). Following a period of dormancy, researchers began re-investigating the potential therapeutic application of MDMA with federal approval, and initial Phase 1 studies demonstrated safety and tolerability (24–26). A number of phase 2 pilot studies were conducted that showed MDMA could be safely administered to participants with chronic PTSD and that established preliminary efficacy (27). Initial phase 3 results suggest that MDMA-assisted therapy (MDMA-AT) may be a highly efficacious treatment for PTSD compared to placebo (28).

MDMA-AT involves a combination of MDMA administration sessions and non-drug psychotherapy sessions delivered by a team of two therapists. Before the first medicine session, the therapists help establish a safe and trusting alliance, provide information about the effects of MDMA, and teach skills for how to best navigate an MDMA experience. During medicine sessions, therapists mostly adopt a non-directive approach that encourages a participant to focus on their inner experience with therapist-participant interaction primarily focused on providing support and reassurance in addition to maintaining safety. MDMA-AT has been posited as an effective treatment for PTSD due to MDMA’s capacity to engender feelings of trust and safety that then facilitate psychotherapeutic processes related to fear extinction and memory reconsolidation (29). In other words, MDMA is thought to promote a context in which participants can talk about and process traumatic memories that have been previously too painful to confront. Therefore, this combination of a drug-induced altered state of consciousness with psychotherapy may offer unique benefits compared to traditional psychotherapeutic or psychopharmacological interventions.

#### 1.1.1. Safety and tolerability of MDMA

MDMA increases activity related to serotonin, norepinephrine, and dopamine (30) as well as oxytocin, vasopressin, prolactin, and cortisol (31–33). Studies of nonhuman animals have demonstrated neurotoxic effects of MDMA, but most of these studies involved larger doses or increased frequencies that make them incomparable to typical human use (34). The recreational use of MDMA or “ecstasy” or “molly” has been linked to toxicity and adverse events including fatalities due to hyperpyrexia, cardiac arrhythmias, and cerebrovascular accidents (35). However, it is difficult to generalize side effects and outcomes from recreational to clinical use because recreational context increases the likelihood of uncontrolled factors like high rates of adulteration (36), combined use with alcohol and other drugs, and environmental factors such as late-night dancing and dehydration. Like other medications that impact the serotonergic system, use of MDMA may also be related to mild or moderate valvular heart disease (37–39). While MDMA has a reputation for impairing cognitive functioning, a study comparing ecstasy users and non-users found no evidence of cognitive impacts associated with its use (40). Finally, while there is some evidence of addiction to MDMA in recreational settings (41), there has been no evidence of abuse in the context in clinical trials (28).

When used in MDMA clinical trials, serious adverse events have been rare (34). In the context of PTSD, common side effects reported on the day of medicine sessions appear relatively minor and included anxiety, jaw clenching, lack of appetite, headache, and fatigue (42). By day 5 following the medicine session, around 20% of participants included mild–moderate anxiety and fatigue, with these symptoms resolving for most by the seventh day follow up. Similarly, in the trial of MDMA-AT for social anxiety among autistic adults (43), 50% of the sample reported mild–moderate AEs during the course of treatment and 75 % of participants reported brief increases in anxiety within the first hour following MDMA administration. MDMA has been shown to increase anxiety transiently among healthy controls and those included in clinical trials (44), but anxiety has generally been found to resolve quickly. We anticipate transient increases in anxiety among participants in this study, particularly given the prevalence of pre-existing anxiety symptoms in our sample. Vitals and AEs will be judiciously monitored to ensure participant safety and tolerability, as detailed in section 2.2.4.

### 1.2. MDMA-AT for SAD

The first published study testing MDMA-AT for social anxiety was a randomized, placebo-controlled double-blind pilot trial of MDMA-assisted therapy for social anxiety in autistic adults (43). Participants (*N =* 12) were randomly assigned to receive MDMA-AT (*n* = 8) or an inactive placebo (*n* = 4). The structure of the therapy consisted of two experimental drug sessions (MDMA or placebo) spaced approximately 1-month apart, with each experimental session preceded by three weekly 60 to 90-min non-drug preparation sessions and followed by three weekly 60 to 90-min non-drug integration sessions. Non-drug therapy sessions included standardized mindfulness-based approaches adapted from dialectical behavior therapy and aimed to support participants struggling with interpersonal relationships, emotion regulation, and distress tolerance. Changes in social anxiety were measured by assessing participants’ scores on the Liebowitz Social Anxiety Scale (45) at baseline, 1 month after the second medicine session, and at the six-month follow-up.

Results indicated that participants in the MDMA-AT group experienced significantly greater reductions in social anxiety symptom severity compared to the placebo group immediately following treatment [*d* = 1.4, 95% CI (0.07, 2.87)] with an enduring large effect at the six-month follow-up assessment [*d* = 1.1, 95% CI (0.31, 2.52)]. In addition, qualitative reports from participants who completed the full course of MDMA-AT (*n* = 7) suggested a range of positive interpersonal outcomes including increased confidence in school, work, and in relationships with friends, family, and romantic partners, initiating of dating for the first time (*n* = 2), and feeling freer to explore and express questions of gender identity (*n =* 2).

Although autism spectrum disorder appears to confer greater risk of developing SAD relative to the general population, clinically elevated levels of social anxiety are found only in the minority of autistic individuals, signifying that SAD is a problem distinct from autism itself despite having some features in common (46). As such, results reported by Danforth et al. (43) indicate the need for further study of the feasibility and effectiveness of MDMA-AT for the treatment of SAD in the absence of autism.

### 1.3. Possible advantages of MDMA-AT compared to treatment as usual for SAD

While data is lacking comparing MDMA-AT to treatment as usual (TAU) for social anxiety disorder, research on MDMA-AT in the context of PTSD may provide some useful guidance. The two FDA-approved pharmacotherapies for PTSD, paroxetine and sertraline, are also two of the three FDA-approved pharmacotherapies for SAD (along with extended-release venlafaxine). A recent review (44) comparing aggregated results from participants in six phase-two trials of MDMA-AT for PTSD with sponsor-initiated phase-three trials of the current FDA approved pharmacotherapies for PTSD, paroxetine and sertraline, registered in the FDA drug database, found that MDMA-AT was associated with larger reductions in PTSD symptom severity measured *via* the Clinician-Administered PTSD Scale (4th edition; MDMA-AT: *d* = 0.9) compared to either paroxetine (*d* = 0.45–0.56) or sertraline (*d* = 0.31–0.37). Participants receiving active MDMA-AT compared to active SSRI treatment also reported fewer adverse events from baseline to primary endpoints of the trials, and lower dropout rates (6.7% for MDMA-AT, 11.7% for paroxetine, and 28% for sertraline). Future research will be needed to see if these patterns related to treatment efficacy and tolerability replicate in the SAD population.

One explanation for potentially enhanced tolerability relative to SSRI treatment is the different treatment course and setting involved in MDMA-AT. With the administration of MDMA in single doses spaced 1 month apart in a controlled setting, adverse drug effects are more likely to occur on the day of the medicine session and dissipate over the next few days, while daily SSRI may be more likely to result in prolonged adverse drug effects due to chronic administration. Patient adherence to drug administration and tapering recommendations are also less of an issue in MDMA-AT than with SSRIs, as doses are not patient-administered. Though research is needed to examine whether efficacy outcomes extend to patients with SAD, it is plausible that the safety and tolerability of MDMA-AT for PTSD relative to pharmacotherapy would translate.

Regarding psychotherapy for SAD, MDMA-AT may provide an effective and viable treatment option for those who do not respond or do not prefer existing psychotherapies. Though exposure-based CBT is largely effective in reducing social anxiety symptoms, results from a meta-analysis analyzing treatment outcome data for CBT for adult anxiety disorders found that only 40% of participants in social anxiety samples (k = 11) met criteria for remission of the disorder at post-treatment (17).This suggests that although CBT helped significantly reduce social anxiety symptoms in these samples, a majority of participants still reported clinically significant distress or impairment following therapy, signifying room for greater benefit. Similarly, approximately 18% individuals drop out of exposure therapy for SAD (18), indicating a sizeable minority of clients do not receive the full intended dose of treatment. One possible benefit of MDMA in the context of psychotherapy is its potential to create feelings of safety that may allow people to approach difficult therapeutic material with more ease (29), potentially reducing dropout. If this were the case, MDMA-AT may be a viable alternative to individuals who, for whatever reason, feel unable or do not prefer to engage in traditional exposure therapy. Research is needed to test these ideas.

### 1.4. Possible processes of change in MDMA-AT for SAD

Though research examining the effectiveness of MDMA-AT for various indications has grown rapidly in the last decade, few studies have measured therapeutic processes of change that account for the MDMA-AT’s effectiveness. As a result, our understanding of *how* MDMA-AT facilitates healing remains poorly understood. To date, one randomized controlled trial (RCT) of MDMA-AT for PTSD examined trait measures of openness to experience and neuroticism to examine whether MDMA-AT may account for enduring shifts in these personality traits as they were associated with treatment outcomes (47). Results indicated that MDMA-AT for PTSD resulted in larger changes in openness to experience, and that those who had the greatest increase in openness to experience reported greater decreases in PTSD symptoms. Authors argued that MDMA-AT may have facilitated an increase in the enduring personality change of the tendency to seek out new experiences and openly reflect on life experiences, which might have reduced PTSD symptoms.

The advancement of scientific progress in studying MDMA-AT depends on examining unique theory-driven processes of change hypothesized to occur during treatment. Understanding processes of change, and not just efficacy outcomes, can inform clinicians’ and researchers’ abilities to tailor the psychotherapy component of the intervention, thereby potentially improving clinical outcomes (48). As we are not aware of any published studies reporting quantitative data on psychological change processes of MDMA-AT for social anxiety disorder, we outline several theory-driven processes of change relating to MDMA-AT for SAD to advance this aim. The processes outlined in this section directly inform the assessments outlined in the trial protocol described below.

MDMA has been posited as a potentially potent treatment for psychological problems involving social dysfunction, such as social anxiety disorder, major depressive disorder, and autism, due to its effects on social perception and cognition, as well as on hormones implicated in interpersonal bonding such as oxytocin & prolactin (49). Though data available from clinical trials are lacking, we have published two comprehensive reviews of correlational and experimental data that outline several psychological processes of change^1^ that may operate in MDMA-AT for SAD to facilitate healing and improve social functioning (51, 52). Though a detailed review is outside the scope of this paper, we briefly review relevant change processes to frame how these are measured in this clinical trial.

#### 1.4.1. In-session processes of change

Based on research demonstrating that the effects of MDMA are dependent on the social environment in which it is taken (53, 54), theoretical analysis of processes of change relevant to MDMA-AT for SAD has been divided into processes occurring proximally within the medicine session itself (51) and enduring processes that could disrupt maintaining factors of SAD over the longer-term (52).^2^

Regarding processes that may occur in the medicine session itself,^3^ Luoma and colleagues (51) put forth three interdependent processes of change posited to occur in medicine sessions of MDMA-AT for SAD: enhanced memory reconsolidation of core shame memories, a strengthened therapeutic relationship between the client and therapist that creates a safe context for rapid therapeutic change, and the promotion of self-transcendent experiences and emotions that serve to disrupt negative, self-referential process and dysfunctional interpersonal behaviors (2).

Memory reconsolidation has been asserted as a transdiagnostic mechanism of change common to many psychotherapy approaches (55) and has been proposed specifically in the context of MDMA-AT for PTSD (29). Memory reconsolidation refers to a process of neuroplasticity thought to occur when memory is activated and initiates a critical period in which the memory trace is susceptible to modification with new information prior to being consolidated again into long-term memory (55). In the context of SAD, MDMA-AT is hypothesized to facilitate memory reconsolidation of core memories of shame, ostracism, devaluation, or rejection frequently reported by people with SAD (2) that function to maintain the core fear in SAD: that the authentic self is flawed and revealing it to others will result in rejection. As MDMA frequently elicits experiences of authenticity (21, 56), medicine sessions are hypothesized to activate the core fear and relevant shame memory traces and intensify prediction errors that could update the memory trace through facilitating experiences of peace, safety, and acceptance rather than shame and rejection (for a detailed discussion see (51).

The memory reconsolidation process, as well as other therapeutic interventions, may be strengthened by a therapeutic relationship between clients and their therapists facilitated by MDMA. MDMA is posited to strengthen the therapeutic alliance, particularly the therapist-patient bond, by increasing feelings of safety and acceptance (57) and reducing clients’ social threat sensitivity (56), thereby reducing the need for safety behaviors that normally disrupt or prolong the process of creating emotional intimacy for people with SAD. Through the lens of inhibitory learning theory, 8-h MDMA sessions where clients repeatedly behave authentically and are met with safety from the therapists, provide a prolonged context for expectancy violations to occur and new learning to solidify (58). This may be augmented as a consequence of reduced amygdala activity during MDMA administration. Applied research has shown that MDMA acutely decreases activity in the amygdala (59), and there is some indication that MDMA may decrease reactivity to harsh faces (60) and increase responses to happy faces (59). This action is compatible with its reported reduction in fear or defensiveness, and is in contrast to the stimulation of the amygdala observed in animal models of conditioned fear (61, 62). MDMA also promotes playful, affiliative feelings toward others (63), increased use of social or relational words (64, 65) and may promote prosocial and warm expressions from clients toward their therapist thereby triggering greater reciprocal expression of affiliative emotions from the therapists (66). This reciprocal process could potentiate new, corrective interpersonal and emotional learning that could generalize to other relationships in the client’s life.

Finally, MDMA-AT may also help address problems related to the self that are characteristic of SAD (67–71). Though many conceptualizations of self-exist, one common distinction includes the separation between the self-as-content [or self-as-object (69); or conceptualized self (72); also referred to as “me” and the self- as-subject (69), or the observer self (72); also described as “I”]. Most models of social anxiety disorder focus on the content of the self, with literature indicating that self-related thoughts and images among people with SAD are overwhelmingly negative and rooted in shame. Research examining the subjective sense of self (“I”) in SAD is more limited, with the study of attentional processes as the notable exception. Research shows that attentional processes of people with SAD tend to be highly self-referential and are characterized by increased attention toward threat-related social cues (73) and difficulty attending to positive and affiliative cues from others (74). Taken together, these processes related to self-conceptualization are thought to serve as barriers to socially contingent responding that is necessary in forming warm, authentic relationships with others. MDMA may help address some of these self-related difficulties through fostering experiences with self-transcendence. MDMA administration may elicit strong self-transcendent positive emotions such as compassion, awe, gratitude, inspiration, and love, that have been shown to increase perceptions of social connectedness and increase prosocial behavior that could potentially decrease the rigid, self-critical and self-focused processing normally dominating the attention of individuals with SAD (for a detailed discussion, see (51).

#### 1.4.2. Enduring processes of change

Randomized controlled trials of MDMA-AT for PTSD and social anxiety among Autistic adults have demonstrated maintained symptom reduction from two to 6 months following the primary outcome assessment (75), suggesting persistent, potentially trait-level changes among study participants who received MDMA-AT. Luoma and Lear (52) argued that MDMA-AT has strong potential for disrupting underlying affective, cognitive, behavioral, and neurological structures that maintain social anxiety and outlined four possible enduring processes of change that may result in long-term improvement in social functioning weeks or months following medicine sessions. Specifically, MDMA-AT may (1) reduce social anhedonia and enhance social reward sensitivity, (2) reduce heightened social threat sensitivity and foster an increased sense of safety in social situations, (3) foster acceptance of shame and reduction of self-criticism through the generation of self-transcendent emotions, and (4) decrease dysfunctional social behaviors, such as submissive behavior and expressive suppression, thereby increasing intimacy with others and improving social relationships (52).

##### 1.4.2.1. Social anhedonia and social reward

Many people with SAD experience social anhedonia (76), a term used to describe the reduction in positive affect in response to social situations and infrequent social approach behaviors observed in SAD. SAD is characterized by low levels of positive affect and difficulty experiencing affiliative emotions in response to social situations paired with elevated levels of negative affect in response to social rejection and increased sensitivity to social punishment (76–78). Taken together, this pattern is thought to bias people with SAD away from social approach goals related to seeking connection and toward avoidance goals related to reducing social threat (69, 79). Shifting the relative balance of approach versus avoidance goals may promote recovery from SAD. MDMA-AT may help make social interactions more rewarding and less punishing both during and after the treatment course and thereby shift the balance of approach versus avoidance goals. MDMA has been shown across many studies to affect brain systems and hormones related to social bonding and positive social reinforcement (49, 81) and may increase motivation to connect with others even after the medicine session. Subjectively, ingestion of MDMA is associated with people reporting increased feelings of love, peace, and safety (81), and a subjective sense of wanting to be with others (82). A recent series of studies with mice indicate that the effects of MDMA on social behavior likely extend beyond the context of acute medicine, and crucially may re-open a critical window for social reinforcement among adult mice that otherwise disappears with age for more detail see (54). Another series of mouse studies also demonstrated that after multiple MDMA administrations, mice showed increased social behavior toward unfamiliar mice, with the effect being greater when they ingested MDMA in the presence of another mouse (83). Considered together, these findings suggest that MDMA may enhance motivation to connect with others, increase the likelihood that people will find social interactions with strangers’ rewarding rather than punishing, and that these effects may extend beyond medicine sessions into the person’s social context. It is possible that temporary increases in the reinforcement value of social interactions could initiate a new pattern of behavior in which individuals with SAD seek out previously avoided social situations and have more frequent positive social experiences, resulting in a positive chain of events in which affiliative behaviors toward others elicits reciprocal affiliative behaviors toward oneself [e.g., (84)], thereby altering the ratio of approach- vs. avoidance-focused social goals that appear to maintain SAD.

##### 1.4.2.2. Heightened social threat

A heightened tendency to experience social stimuli as threatening has been posited to be the central mechanism in the development and maintenance of SAD (2). People diagnosed with SAD show heightened amygdala activity to stimuli related to social evaluative threat, such as negative emotional expressions or critical comments about oneself (85–87). By contrast, MDMA administration among healthy volunteers is associated with decreased amygdala activity in response to social threat (59) and reduced self-reported negative reactions to social rejection (88), suggesting promise in its ability to therapeutically alter this process in people affected by SAD.

The ability to feel safe around others and respond to social interactions in a fluid and contingent manner appears to be mediated through the same neuroanatomical system corresponding with a felt sense of safety, termed the social engagement or social safety system. The social safety system refers to the functioning of the parasympathetic nervous system, more centrally the vagus nerve and ventral vagal complex, and is thought to be indexed by variation in high-frequency heart rate variability [HF-HRV; (89, 90)]. Research suggests chronic under activation of the vagal brake in individuals with SAD as demonstrated by lower HF-HRV (measured through respiratory sinus arrhythmia or RSA) at rest compared to healthy controls (91). Though it is unclear whether alterations in vagal pathways play a role in the development of SAD or are better conceptualized as SAD sequelae, people with social anxiety disorder display a rigid response style to social situations that involves the inability to inhibit threat-based responses in non-threatening situations (65). Thus, improvements in SAD are likely to be dependent upon the extent to which people experience a greater sense of safety in social encounters.

It has been hypothesized that the increased feelings of love and sociability often reported by individuals who have ingested MDMA may be due to increased parasympathetic activity secondary to MDMA (88). Preliminary results have failed to find support that for this hypothesis, with two studies reporting that MDMA is associated with decreased activation of the parasympathetic nervous system, and inconsistent findings on the relation between subjective effectives and indices of parasympathetic activity (92, 93). However, it remains unclear how parasympathetic activity may change over time following MDMA ingestion, particularly when supplemented with therapy. Anecdotal reports of early therapists using MDMA with clients, posited that MDMA-AT may potentiate an enduring change in social threat perception in SAD by helping people feel safer with others and clients often reported greater ease in relating to others in both close and more distant relationships for weeks or months afterwards (21). MDMA may result in changes in social safety by facilitating a greater focus on cues related to affiliation and intimacy in social situations as MDMA has been shown to promote responding to positive social cues preferentially (60), suggesting MDMA-AT could bolster internally experienced safety by shifting clients’ focus toward affiliative aspects of social experiences (e.g., being accepted and feeling connected).

##### 1.4.2.3. Shame and shame-related coping

Many clinical and etiological models of SAD point to the significance of shame in maintaining heightened social threat perception in SAD (94). Research shows that negative self-representations, negative interpretation biases, negative self-imagery, and social evaluative thoughts characteristic of shame are observed at higher levels among individuals with SAD compared to healthy controls [see (2) for a review]. Some clinical models of SAD hold that the anxiety experienced by people with SAD is a secondary response to the anticipation of exposing the core shameful self to others (71, 95). In this view, SAD is maintained because individuals with SAD develop a range of shame avoidance strategies that function to protect them from experiencing rejection, ostracism, and shame that may confirm predictions based on shame-based self-referential thinking (e.g., “I’m going to look stupid”).

Self-criticism is a key internal shame-avoidance strategy in SAD (96, 97) that appears to be applied frequently and inflexibly around social interactions (97). This inflexible pattern of self-criticism is thought to perpetuate anxiety in social situations by reinforcing beliefs of low social rank and inferiority (98) as well as triggering defensive arousal associated with a sense of social threat. MDMA-AT reduces shame in people with SAD by facilitating experiences of compassion or kindness toward the self through neurochemical changes, as well as through modeling from the therapist in medicine sessions. Regarding the former, two experimental studies with ecstasy (presumably containing MDMA in one study and tested to confirm MDMA in the other) demonstrated that ecstasy increases feelings of self-compassion and reduces self-criticism (99, 100). Regarding the relationship with the therapist, MDMA-AT also appears to strengthen the bond between therapist and client and boost feelings of safety in medicine sessions, potentially laying the foundation for replicating these types of safe interactions with others following participation in MDMA-AT (101, 102). Furthermore, the tendency for MDMA to strengthen emotional responses to positive social stimuli (82, 103) is likely to make therapists’ responses of compassion and warmth more salient at a visceral level. This may potentially enhance a prediction error when the person reveals their authentic (and seemingly flawed) self in the therapy session when it is met with acceptance and caring rather than the expected rejection or ridicule from their therapist. Ultimately, this could enhance memory reconsolidation of shameful memories contributing to the person’s core sense of shame (51).

##### 1.4.2.4. Dysfunctional social behavior

People with social anxiety frequently rely on interpersonal behaviors that function to decrease their anxiety in social scenarios, yet simultaneously evoke discomfort in and desires for social distance from others (2). These behaviors, often called “safety behaviors” are hypothesized to maintain SAD by eliciting responses from other people that reinforce the person’s perceptions of social inadequacy, deficiency, and low social status [e.g., (104), and interfere with developing and maintaining close and secure relationships (105, 106) see (2) for a review]. These interpersonal responses also lead to subjective feelings of inauthenticity and perceptions from others that the person is not being genuine among people with SAD (107, 108). Authenticity refers to whether a person is behaving in a way that is true to what they really experience (109) and has been associated with higher personal (110–112) and interpersonal well-being (113, 114). Several studies suggest that reductions in safety behaviors and increases in authentic interpersonal expression may play an important role in recovery from SAD [e.g., (115, 116)]. People taking MDMA often report feeling more authentic (56), while the increased perceptions of safety and reduced shame due to MDMA-AT could presumably result in a decrease in safety behaviors and improvements in social functioning. A qualitative study of people in MDMA-AT for PTSD (57) found that 12 out of 19 of the interviewees reported subjective improvements in relationships and social skills. Similarly, the novel social behavior previously described in Danforth et al.’s (43) study among some of the autistic adults who received MDMA, such as the initiation of dating and authentic exploration of gender identity, suggest notable shifts in social behavior as a consequence of MDMA-AT.

## 2. Study protocol: social anxiety MDMA-assisted therapy investigation

### 2.1. Overview

Below, we describe the study protocol of the Social Anxiety MDMA-Assisted Therapy Investigation (SAMATI; clinicaltrials.gov/show/NCT05138068), an investigator-initiated phase two, open-label, delayed treatment randomized controlled trial examining MDMA-AT for SAD. We chose this design to maximize our ability to obtain more data on MDMA-AT at lower cost than a placebo-controlled design. To our knowledge, this is the first proposed pilot trial of MDMA-AT for SAD as an indication outside the context of autism. The study aims to obtain an estimate of effect size for MDMA-AT for SAD on measures of social anxiety, functional outcomes, and potential therapeutic processes of change, as well as evaluate safety and feasibility. By evaluating the effectiveness and acceptability of the intervention among a small group of participants, we hope results from this study can inform the methods utilized and hypotheses studied in larger-scale RCTs, thereby improving the resource efficiency and replicability of future studies. To that end, a secondary aim is to develop a theory-driven manual for MDMA-AT for SAD that can be used in future clinical and research designs.

### 2.2. Methods and analysis

#### 2.2.1. Recruitment and selection

Twenty participants with a DSM-5 diagnosis of SAD will be recruited for enrollment over a two-year period. Participants may be recruited *via* online and print advertising, social media campaigns, public awareness campaigns, flyers, and communication with local health care providers and centers. In the initial phase of recruitment, communication with local health care systems was focused on providers or organizations that represent or have contact with historically oppressed or marginalized populations to provide opportunities for enrollment by those more likely to be excluded from clinical trial research traditionally. People in these communities are also at elevated risk for internalizing negative self-referential beliefs characteristic of SAD as a consequence of experiencing stigmatization, prejudice, and discrimination due to structural racism and unjust dominant cultural systems (117).

Completion is defined as undergoing the final assessment visit during which the primary outcome is measured. Publicly available inclusion and exclusion criteria^4^ for the study can be found in Table 1.

**Table 1:** Inclusion and exclusion criteria.

Inclusion criteria
Between 18 and 65 years old
Fluent in speaking and reading English
Able to swallow pills
Agree to have study visits recorded, including Experimental Sessions, assessments, and non-drug psychotherapy sessions
Provide a contact (relative, spouse, close friend or other support person) who is willing and able to be reached by investigators in the event of a participant becoming suicidal or unreachable
Agree to inform investigators within 48 h of any medical conditions and procedures
If able to become pregnant, must have a negative pregnancy test at study entry and prior to each Experimental Session, and must agree to use adequate birth control through 10 days after last Experimental Session
Agree to necessary lifestyle modifications
Able to identify support person who can stay with participant overnight after Experimental Sessions
Has a suitable home environment to allow completion of all study procedures, including sufficient privacy and access to computer or mobile device with internet access
At Screening, meet DSM-5 criteria for current Social Anxiety Disorder, generalized subtype
At Screening, may have well-controlled hypertension that has been successfully treated with anti-hypertensive medicines, if they pass additional screening to rule out underlying cardiovascular disease
At Screening, may have asymptomatic hepatitis C (HCV) that has previously undergone evaluation and treatment as needed
At Enrollment confirmation for those in delayed treatment group, continue to meet criteria for Social Anxiety Disorder, generalized subtype
Enrollment is allowed with glaucoma only with the approval of their ophthalmologist
Exclusion criteria
Are not able to give adequate informed consent
Are currently engaged in compensation litigation whereby financial gain would be achieved from prolonged symptoms of Social Anxiety Disorder or any other psychiatric disorder
Are likely, in the investigator’s opinion and via observation during the Preparatory Period, to lack social support or lack a stable living situation or supportive family/network
Have any current problem which, in the opinion of the investigator or study physician, might interfere with participation
Would present a serious risk to others as assessed by investigator, study physician, or study team
Require certain excluded medications
Have evidence or history of significant (controlled or uncontrolled) hematological, endocrine, cerebrovascular, cardiovascular, coronary, pulmonary, renal, gastrointestinal, immunocompromising, or neurological disease, including seizure disorder, or any other medical disorder judged by the investigator to significantly increase the risk of MDMA administration (participants with hypothyroidism who are on adequate and stable thyroid replacement will not be excluded)
Have uncontrolled hypertension using the standard criteria of the American Heart Association (values of 140/90 millimeters of Mercury [mmHg] or higher assessed on three separate occasions)
Have a marked baseline prolongation of QT/QTc interval
Have a history of additional risk factors for Torsade de pointes (e.g., heart failure, hypokalemia, family history of Long QT Syndrome)
Require use of concomitant medications that prolong the QT/QTc interval during Experimental Sessions
Have symptomatic liver disease
Have history of hyponatremia or hyperthermia
Weigh less than 48 kilograms (kg)
Are pregnant, nursing, or are able to become pregnant and are not practicing an effective means of birth control

Some eligibility criteria have been omitted to protect the integrity of the recruitment process. Full exclusion/inclusion criteria will be reported with results when recruitment is complete.

#### 2.2.2. Screening and consent

The screening process is divided into two stages: pre-screening and screening. The pre-screening stage features a variety of assessments and meetings with study team members, including multiple online surveys, phone calls, and video calls. If a participant meets eligibility criteria during these initial pre-screening steps, they are invited to an informed consent meeting to discuss the study in more detail and answer any remaining questions they may have before confirming their interest in proceeding to the next, more formal stage of the screening process.

The screening stage involves online surveys, structured clinical interviews with an independent assessor, a review of individual’s medical records, an examination by a study physician, and the completion of drug, pregnancy, electrocardiogram, and other laboratory tests. If an individual meets eligibility throughout both the prescreening and screening stages, the study team reviews all information gathered throughout the screening process to re-confirm enrollment eligibility before inviting the individual to an enrollment meeting. In this enrollment meeting, the person is formally offered the opportunity to confirm their interest in and availability to continue to the preparation period.

#### 2.2.3. Overview of study visits

**Figure 1:**
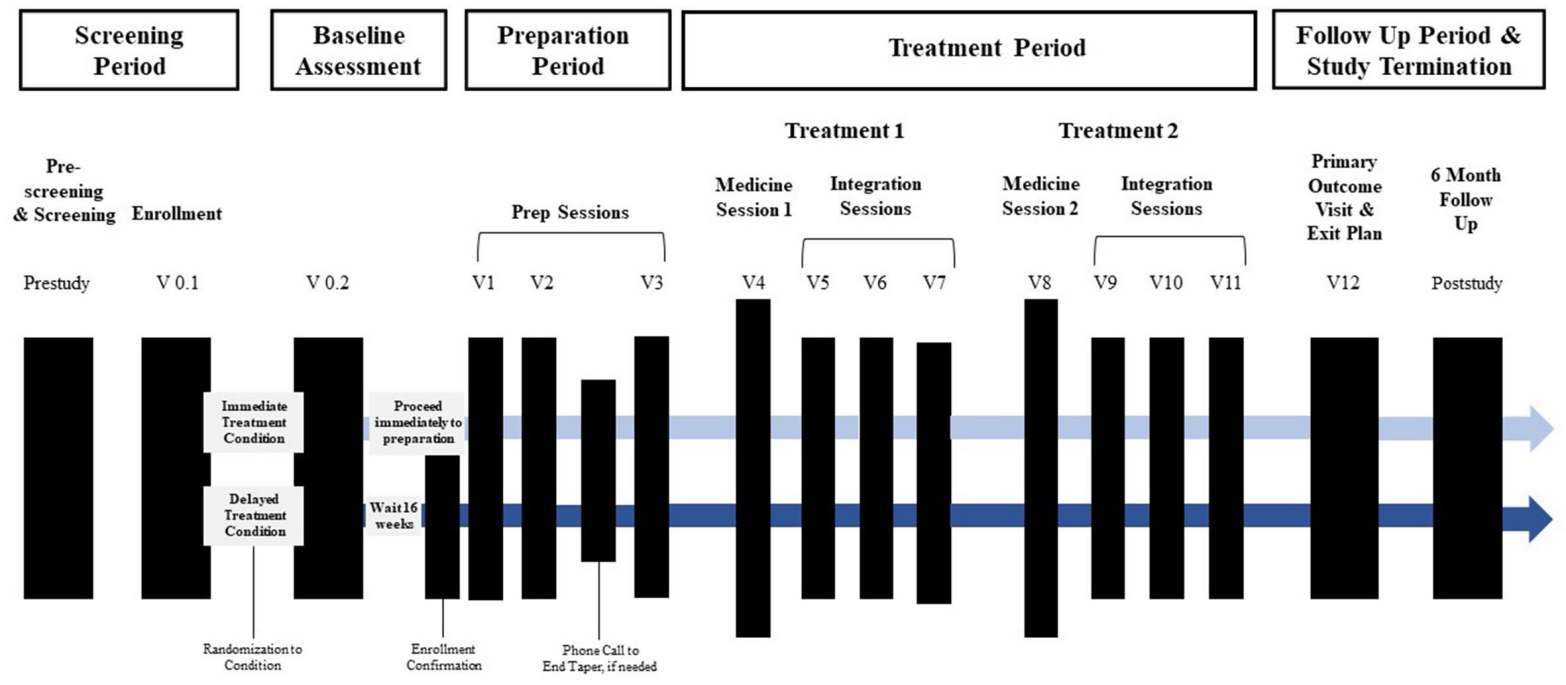
Overview of study timeline. Active treatment sessions (prep, medicine, and integration sessions) begin within 12 days of enrollment confirmation for immediate treatment condition and 16 weeks later for the delayed treatment condition Prep sessions and the first medicine session occur approximately 1-week apart. Integration sessions begin the morning after each medicine session and occur approximately week apart. The primary outcome occurs approximately 2 weeks after the final integration session.

The timeline of study visits can be found Figure 1. Following confirmation of enrollment eligibility, participants are randomized to either the immediate (*n =* 10) or delayed (*n* = 10) treatment condition, and medication tapering is initiated if applicable. Those assigned to the delayed condition engage in monthly phone check-ins with the study team before beginning study procedures after a 16-week delay. For both conditions, the screening and enrollment process is followed by a baseline assessment, preparation period, treatment period, follow-up period, and study termination. In total, the active treatment component includes three preparation sessions, two medicine sessions, and six integration sessions.

##### 2.2.3.1. Assessment visits

Participants complete a series of remote and in-person assessment visits once they are enrolled. The baseline and primary outcome assessments are largely similar. Participants attend a meeting in person in which they complete surveys and participate in a behavioral task where heart rate variability is assessed. They also complete a remote meeting in which they participate in a clinical interview battery that includes measures of social anxiety symptom severity, functional impairment, and safety. Social anxiety symptom severity is also assessed following the second preparation session and at the 6-month follow-up visit.

##### 2.2.3.2. MDMA-AT visits

The general structure of the intervention is adapted from the MDMA-AT for PTSD treatment protocol developed by MAPS (118). Each participant is paired with a team of two therapists. The preparation phase of therapy consists of three 90-min, non-drug preparation sessions, some of which may be conducted remotely *via* telehealth. The aim of these sessions is to build therapeutic alliance(s), educate participants about social anxiety disorder and MDMA, obtain background information related to the participant’s social anxiety, train participants in relevant skills to use during medicine sessions, address any questions or concerns participants may have, and promote a positive set and setting for the medicine sessions.

Medicine sessions occur in-person and last about 8 h. Prior to drug administration, participants acclimate to the study environment, have additional drug and pregnancy urine testing, complete assessments, and have their vitals taken. In the first medicine session, the initial dose of MDMA is 80 mg. In the second medicine session, the initial dose of MDMA is increased to 120 mg unless contraindicated. In both medicine sessions, the participant is administered a supplemental dose of MDMA 40 mg about 1.5 to 2 h after the initial dose is given unless contraindicated. After each medicine session, the participant returns to the study site the following morning to have their first of three integration sessions. Integration sessions are 90-min non-drug psychotherapy sessions that serve to assist the participant with processing their experience during the medicine session and other therapeutic tasks such as implementing behavioral change. The second integration session occurs within 2 weeks of the first, and the third integration session takes place within three to 5 weeks. The second medicine session is scheduled to take place about a month after the first medicine session and is followed by three more integration sessions. The primary endpoint assessment occurs 2 weeks after the last integration session and involves questionnaires and in-person tasks as well as the development of an exit plan. Lastly, the participant is offered the option to take part in a 24-month follow-up post-study.

#### 2.2.4. Safety monitoring and support person involvement

Maintaining and monitoring safety is a primary objective throughout the study. Safety monitoring includes frequent assessments of participant suicidality and self-harm, measuring heart rate, blood pressure, and blood oxygenation during medicine sessions, 24-h on-call availability of study team physicians and therapists, the option of overnight stays if necessary, careful adverse event (AE) monitoring, medical screening, and proper drug storage and management procedures. Monitoring of AEs begins immediately following enrollment and continues throughout the duration of the study.

Additionally, each participant is asked to identify a support person who can provide transportation and stay with the participant overnight following medicine sessions. The therapy team meets with the participant and their support person during the preparation phase in order to assess suitability of the support person and provide basic information about MDMA-AT. The support person is informed that their role is to support the participant’s basic needs and safety by preparing the participant’s food, refraining from drugs or alcohol, being emotionally and physically present, and avoiding taking on a therapeutic role.

At the end of the medicine sessions, the study team and participant collaboratively decide whether it is in the participant’s best interest to leave the study site with their support person or if it would be best for the participant to remain at the study site overnight with a night attendant. Assessment of suicidality and self-harm as well as measurement of vitals toward the end of the session helps to inform this decision. If there is a medical or psychiatric emergency, the study team will evaluate whether the participant should be referred to the nearest emergency room.

#### 2.2.5. Therapist training and supervision

All study therapists received a certificate of completion from the MAPS MDMA Therapy Training Program. This training provides instruction for conducting MDMA-AT for PTSD and totals over 100 h including attending retreats, presentations, discussion groups, as well as completing coursework and assignments. Supervision, including review of video recordings of sessions, will be ongoing throughout the study, some of which will be provided by a MAPS-approved supervisor.

#### 2.2.6. Manual development

The therapist manual to guide preparation, medicine, and integration sessions is currently being developed by the study therapists and principal investigator. The therapist manual is informed by the MAPS MDMA-AT for PTSD treatment manual but includes significant modifications. First, the current treatment manual focuses on social anxiety disorder as the indication rather than PTSD. Second, the current treatment manual is more heavily rooted in evidence-based psychotherapy methods including Acceptance and Commitment Therapy [ACT; (119) and Emotion-Focused Therapy (68)], rather than the more eclectic approach used in the MAPS PTSD trials. It also incorporates elements based on the hypothesized processes of change outlined earlier. Additionally, the treatment manual is more explicit in addressing diversity in identities of participants and how to deliver culturally appropriate care. Guidelines for the ethical and appropriate use of therapeutic touch are outlined, as well as guidelines for obtaining informed consent around touch. Modifications to the treatment manual are expected to occur as a result of supervision, video review, ongoing reflection, and feedback so as to have a more well developed, comprehensive treatment manual by the time the study is completed so it can be tested in future trials.

#### 2.2.7. Study measures

##### 2.2.7.1. Primary outcome

###### 2.2.7.1.1. Liebowitz social anxiety scale

The primary outcome measure for the study is the clinician-administered Liebowitz Social Anxiety Scale [LSAS; (120)]. The LSAS is a 24-item, semi-structured interview assessing social anxiety disorder symptoms that has been widely used in clinical studies, including in previous research on the use of MDMA-AT for social anxiety in autistic adults (43). The LSAS has been shown to have good internal consistency (score inter-correlations ranging from 0.81 to 0.96), reliability, convergent validity, and sensitivity to treatment (120).

###### 2.2.7.1.2. Blinding and minimization of bias

To minimize bias in measuring effect, the LSAS will be administered by independent raters (IR) *via* live video interviews. The IRs are blinded to full study design, visit number, and any data from the treating therapy team after baseline. IR visits are assigned based on a combination of availability and attempts to maximize blinding by minimizing the number of assessments by the same IR.

##### 2.2.7.2. Secondary outcomes

The Sheehan Disability Scale [SDS; (121)] is used to assess degree of impairment in domains of work/school, social life, and home life. The Internalized Shame Scale [ISS; (122)] is used to assess changes in shame as a result of the intervention. The Acceptance of Shame and Embarrassment Scale [ASES; (123)] is used to measure changes in shame-regulation, specifically participants’ acceptance and experiential avoidance of shame and embarrassment. Changes in perceptions of belonging as a result of treatment are examined using the Thwarted Belongingness subscale from the Interpersonal Needs Questionnaire [INQ; (124)]. The Hidden Self Scale [HSS; (125)] is a 4-item subscale of the Core Extrusion Schema–Revised that is used to measure changes in self-concealment as a result of the intervention. Finally, the Self-Compassion Scale-Short Form [SCS-SF; (126)] will be used to investigate shifts in self-compassion.

##### 2.2.7.3. Behavioral and other exploratory outcomes

###### 2.2.7.3.1. Behavioral task

Research indicates that conversations involving greater self-disclosure are associated with increased displays of dysfunctional social behavior compared to conversations requiring less self-disclosure in SAD samples (127). Therefore, to assess social anxiety, participants will engage in a brief closeness-generating experimental task with a stranger [Fast Friends task; (128)] at baseline and primary outcome assessment points. Participants’ heart rate will be assessed during the task to examine changes in high-frequency heart rate variability (HF-HRV). HF-HRV is a measure of peripheral parasympathetic nervous system activity that has been shown to be associated with a wide range of positive health care and social outcomes (129, 130).

###### 2.2.7.3.2. Memory interview

A modified version of the Waterloo Images and Memories Interview [WIMI; (131)] is being used to assess changes in SAD-related autobiographical memories. The WIMI has primarily been used in studies of memory rescripting interventions for social anxiety disorder, which utilize imagery to attempt to change the structure and function of autobiographical memories believed to be central in maintaining negative imagery typical of social anxiety disorder (132).

###### 2.2.7.3.3. Daily diary assessments

Participants will complete brief daily diary surveys for the 7 days preceding and the 7 days following each medicine session to examine acute changes in process variables posited to be affected by MDMA administration. Participants complete one 5-min survey each evening. Daily diary items were piloted in a previous study conducted by the lab on Amazon’s Mechanical Turk (preregistered atosf.io/s6bwg/).

###### 2.2.7.3.4. Reaction to touch measure

Touch between therapist and client commonly occurs in MDMA-AT and has been discussed as an important element of therapeutic benefit (118). Applied research has shown that MDMA enhances people’s perceptions of the pleasantness of social touch relative to other psychoactive stimulants, and this has been posited as one means through which MDMA may positively bias social interactions [e.g., (122)]. Examining reactions to social touch is also particularly relevant in the context of SAD. People with elevated social anxiety have been reported to experience greater avoidance of social touch and increases in anxiety, self-consciousness and embarrassment, and avoidance in response to touch compared to their less anxious counterparts (133, 134).

In addition, the use of touch is controversial and there is little data on how the use of touch in psychotherapy is related to treatment outcomes or experienced by clients. Since there are currently no established measures to gather data on reactions to physical touch in therapy and to better understand its role in MDMA-AT, an 18-item measure was created by the study team to assess participant’s perceived benefits, feelings of safety, control, and discomfort with touch during medicine sessions (measure and associated publications available at osf.io/qm7bk). This measure will be administered the day after each medicine session.

###### 2.2.7.3.5. Post-medicine session interview

Participants will complete a semi-structured, qualitative interview after each medicine session to gather information about their experience. The interview will focus on gathering information about challenging, beneficial, and interfering or iatrogenic experiences.

#### 2.2.8. General analysis strategy

##### 2.2.8.1. Sample size estimation

Power analysis for this trial was based on the previously published trial of MDMA-assisted therapy for SAD in autistic adults (43). Danforth and colleagues (43) found a between-group placebo-subtracted effect at the primary outcome point of *d =* 1.4. Based on this effect, a sample of 20 participants is consistent with power of 0.88 to detect a statistically significant between-group difference at the primary endpoint in this study. It is possible the use of a delayed treatment control in this study rather than a placebo control may yield larger between-group effect size compared to the previous trial.

##### 2.2.8.2. Efficacy and process outcomes

Assessment time-points were chosen to prioritize measurement of the acute effects of the intervention (over the 12-week duration of the trial) and enduring changes in SAD symptoms attributable to the intervention. For the primary study outcome, social anxiety symptom severity assessed via the LSAS, treatment effects will be established using intent-to-treat (ITT) analyzes comparing mean LSAS scores at the primary outcome point in the immediate treatment condition (*n =* 10) and the 16-week re-assessment in the delayed treatment condition (*n* = 10). A participant is considered eligible for the ITT analyzes if they have completed at least one medicine session and at least one LSAS beyond the baseline assessment. Secondary analyzes using the LSAS will include means-based comparisons from baseline LSAS assessment to primary outcome, and baseline to 6-month follow up across all participants (*N* = 20) after participants in the delayed treatment group have received the MDMA-AT intervention. Analyzes assessing functioning *via* the SDS will be conducted using the same methods.

Treatment effects for all other secondary psychological processes will be assessed for the entire sample (*N* = 20) using a series of paired samples t-tests by comparing scores at baseline, primary outcome, and 6-month follow-up where relevant to assess immediate and sustained changes following the intervention. Pearson correlations between changes in LSAS scores from baseline to primary outcome and changes in secondary psychological process variables will be calculated to examine their relevance as putative therapeutic processes of change. Finally, descriptive statistics for all primary and secondary outcome variables will be reported at each time point.

##### 2.2.8.3. Feasibility and safety outcomes

Recruitment and retention rates will be reported as main indicators of feasibility. A consort diagram will be used to present participant retention at each phase. The rate and pattern of missing data in baseline and outcome measures will also be reported. Safety analyzes will present safety data including exposure to MDMA, adverse events (AEs), concomitant medications, suicidal ideation and behavior, and vital signs overall and by group.

#### 2.2.9. Ethics and dissemination

This is an investigator-initiated and sponsored trial performed in collaboration with the Multidisciplinary Association for Psychedelic Sciences (MAPS) and the Oregon Research Institute Community and Evaluation Services (ORI CES). Study procedures have been reviewed and approved by the WCG Institutional Review Board, United States Food and Drug Administration, and MAPS. All participants in this study will provide their voluntary, written informed consent to participate. The study site, Portland Psychotherapy Clinic, Research, and Training Center, was audited and found in compliance with the United States Drug Enforcement Agency standards. All study staff have undergone Good Clinical Practice (GCP) training.

The results of this study will be published in academic journals and presented in academic and public domains, including at scientific conferences and in the media. Patient confidentiality will be maintained in all dissemination efforts.

## 3. Discussion

In this paper we have outlined the protocol for the first known pilot trial assessing MDMA-AT for social anxiety disorder. Beyond examining the safety and feasibility of MDMA-AT for SAD, we wish to highlight the examination of potential processes of change of MDMA-AT for SAD as a unique strength of this study design. We encourage future researchers to go beyond primary efficacy outcomes and integrate measurements of change processes into future research designs of MDMA-AT. This is crucial in advancing our scientific understanding of how these medicines operate across individuals of varying backgrounds so that we can better adapt the psychotherapy components of these interventions to maximize effectiveness. We hope the knowledge gained from this study and the novel design aspects incorporated will provide a framework for more efficient, nuanced, and efficacious randomized controlled trials in the future.

## Ethics statement

Study procedures have been reviewed and approved by the WCG Institutional Review Board, United States Food and Drug Administration, and MAPS. All participants in this study will provide their voluntary, written informed consent to participate.

## Author contributions

ML: writing original draft, writing review and editing, project administration, conceptualization, methodology, and supervision. SS: writing original draft, writing review and editing, project administration, and methodology. BP: conceptualization, resources, writing original draft, and writing review and editing. CS: conceptualization, methodology, writing review and editing, and supervision. JL: conceptualization, methodology, investigation, resources, writing review and editing, supervision, project administration, and funding acquisition. All authors contributed to the article and approved the submitted version.

## Funding

This research is currently funded by revenue generated internally by the social enterprise model of Portland Psychotherapy Clinic, Research, and Training Center that serves to fund scientific research. 

## Conflict of interest

The authors declare that the research was conducted in the absence of any commercial or financial relationships that could be construed as a potential conflict of interest.

## Publisher’s note

All claims expressed in this article are solely those of the authors and do not necessarily represent those of their affiliated organizations, or those of the publisher, the editors and the reviewers. Any product that may be evaluated in this article, or claim that may be made by its manufacturer, is not guaranteed or endorsed by the publisher.

## Footnotes

^1^Processes of change can be studied at varying levels, ranging from molecular, neurological, to sociological (50). These theoretical analyses primarily focus on processes of change occurring at the psychological level due to their direct relevance in the development of the psychotherapy component of MDMA-AT for SAD.

^2^Authors specify that theory-driven processes of change outlined across these review papers are likely interdependent, reciprocally reinforcing, and occur in potentially simultaneous and variable sequence. They are discussed as discrete processes and referred to in a temporal order (e.g., in the dosing session vs. long-term) for clarity and should not be interpreted as linear or mutually exclusive constructs.

^3^Though the review focuses on events in the dosing session, the authors note that the content of the preparation and integration sessions are also likely to be central in guiding and reinforcing processes that occur in dosing sessions.

^4^Some inclusion criteria are not yet publicly available to protect the integrity of the recruitment process. Full inclusion and exclusion criteria will be reported with results when recruitment is complete.
